# Variations in cochlear duct shape revealed on clinical CT images with an automatic tracing method

**DOI:** 10.1038/s41598-017-16126-6

**Published:** 2017-12-14

**Authors:** Annerie M. A. van der Jagt, Randy K. Kalkman, Jeroen J. Briaire, Berit M. Verbist, Johan H. M. Frijns

**Affiliations:** 1 Department of Otorhinolaryngology, Leiden University Medical Center, Leiden, The Netherlands; 2 Department of Radiology, Leiden University Medical Center, Leiden, The Netherlands; 3 Department of Radiology, Radboud University Medical Center Nijmegen, Nijmegen, The Netherlands; 4 Leiden Institute for Brain and Cognition, Leiden University Medical Center, Leiden, The Netherlands

## Abstract

Cochlear size and morphology vary greatly and may influence the course of a cochlear implant electrode array during insertion and its final intra-cochlear position. Detailed insight into these variations is valuable for characterizing each cochlea and offers the opportunity to study possible correlations with surgical or speech perception outcomes. This study presents an automatic tracing method to assess individual cochlear duct shapes from clinical CT images. On pre-operative CT scans of 479 inner ears the cochlear walls were discriminated by interpolating voxel intensities along radial and perpendicular lines within multiplanar reconstructions at 1 degree intervals from the round window. In all 479 cochleas, the outer wall could be traced automatically up to 720 degrees. The inner wall and floor of the scala tympani in 192 cochleas. The shape of the cochlear walls were modelled using a logarithmic spiral function including an offset value. The vertical trajectories of the scala tympani exhibited a non-monotonous spiral slope with specific regions at risk for CI-related insertion trauma, and three slope categories could be distinguished. This presented automatic tracing method allows the detailed description of cochlear morphology and can be used for both individual and large cohort evaluation of cochlear implant patients.

## Introduction

The human cochlea exhibits considerable interindividual variability in size and morphology^1–5^. This variation influences the final position of the electrode array after performing cochlear implant (CI) surgery and may be important for patient-specific surgical planning^5,6^. Optimal placement of the electrode array to maximize speech recognition should be achievable in all individual cochleas; to accomplish this, increased insight into cochlear morphology is needed.

The human cochlea is a spiral-shaped cavity in the bony labyrinth with the scala tympani (ST) as the targeted compartment for insertion of a CI electrode array. Both the course and final position of the electrode array are influenced by several factors, including cochlear morphology. In relation to cochlear morphology, Van der Marel *et al*. showed that 12% of the variance in angular insertion depth can be explained by cochlear size alone^5^. Escudé *et al*. studied the effect of cochlear size on insertion depth and reported that a 2 mm larger diameter of the basal turn produces a 5.0 mm larger length of the lateral wall^6^. The diameter or circumference of the basal turn is often used as an indicator to describe the size of the cochlea. The diameter of the basal turn, measured from the center of the round window (RW) to the opposite outer cochlear wall, varies between 5.6 and 8.2 mm, and the circumference of the basal turn, often referred to as the cochlear duct length (CDL), varies in length between 20.3 and 24.3 mm^2^. Dimensional variations of the second turn have been limited investigated, even rarely *in vivo*, but it is conceivable that variations at this level also play an important role in the surgical outcome after cochlear implantation, especially with somewhat longer electrode designs.

Other, less frequently described characteristics of the human cochlea relevant for cochlear implantation are its coiling pattern and vertical trajectory. The Archimedean and logarithmic spiral function are most often used to describe the coiled shape of the cochlea^7^. Previous studies from our group confirmed the applicability of the logarithmic function on the shape of the basal turn^5^. Erixon *et al*. found that each cochlea is individually shaped and reported a large variation in coiling characteristics, suggesting that the spiral functions do not fit all individual cochleas^2^. Specifically, the shape and size of the basal turn of the cochlea is influenced by the coiling pattern; early coiling results in a smaller, more compressed cochlea with an earlier increase in the vertical trajectory. The vertical trajectory of the ST exhibits an undulating slope and likely plays a critical role in the occurrence of intra-cochlear trauma during CI surgery. Earlier *ex vivo* studies have demonstrated three high-risk areas for intra-cochlear trauma along this trajectory^1,8^. First at the site of insertion, near the RW, and is related to the surgical procedure of insertion. The second one is located approximately 180–200 degrees from the RW, where the presence of a downward slope of the ST, in many cases, is likely to alter the course of the electrode array. This may expose the osseous spiral lamina (OSL) or basilar membrane (BM) to compression forces. A subsequent steep upward slope will force the tip of the electrode array to press against the floor of the ST and bend upward^8^. These provoked changes in the course of the implant are considered to be associated with insertion trauma. As with size, height, and coiling pattern, large variation exists in the vertical trajectory, and three slope categories were identified based on the anatomical features of this vertical trajectory in micro-CT data in a series of temporal bones^1^. We hypothesize that, if such a detailed account of the vertical trajectory can be obtained *in vivo*, it may be a valuable pre-operative indicator for estimating the risk of insertion trauma.

Here, we present a novel, automatic method for determining the cochlear walls and floor of the ST from multiplanar reconstructions (MPRs) of clinical CT images. The cochlear walls are traced from the RW up to a cochlear angle of 720 degrees, providing the unique opportunity of assessing cochlear morphology *in vivo* up to and including the second turn. The method will be shown to be applicable in a large population of CI candidates.

## Materials and Methods

### Patient population

We analyzed pre-operative CT scans of all patients consecutively implanted at Leiden University Medical Center in the Netherlands between January 2011 and December 2014. Patients with cochlear malformations (n = 14), obliteration of the cochlear lumen by fibrosis or ossification (n = 16), or fenestral and/or cochlear otosclerosis obscuring identification of the RW (n = 4) were excluded. Scans performed at external institutions or with inferior scan quality due to movement artefacts were excluded (n = 39). A total of 479 inner ear CT scans from 242 patients were included in the analysis. Table 1 summarizes the demographic characteristics of the patient population (n = 242).

**Table 1:** Demographic characteristics

Total population (N = 242 patients)		
Age at implantation	Mean, SD (years)	47, 24
Gender	Male: Female	111:131
Etiology of deafness		
*Congenital*	Hereditary	45
	Acquired	12
	Anatomical	3
	Unknown	50
*Acquired*	Meningitis	10
	Meniere’s disease	8
	Trauma	2
	Otosclerosis	1
	Recurrent otitis	1
*Unknown*		110

All methods were carried out in accordance with relevant guidelines and regulations related to retro-spective research with existing data, as stated by our institutional Medical Ethical Committee.

### Image reconstruction and analysis

At Leiden University Medical Center, CI candidates undergo a CT scan of both inner ears prior to implantation to gain insight into the etiology of deafness and feasibility of implantation as part of a standard work-up. The CT scans evaluated in this study were acquired using an Acquilion scanner (Toshiba Medical Systems, Otowara, Japan). MPRs were assembled from these scans and a 3D coordinate system was applied that enables general assessment of the inner ear anatomy^9^. The basis of this coordinate system is formed by the cochlear view perpendicular to the basal turn. A three-dimensional cylindrical system is created by adding a z-axis through the center of the modiolus with its origin at the helicotrema. The RW is chosen as the zero degree angle of rotation.

### Automatic tracing procedure

**Figure 1:**
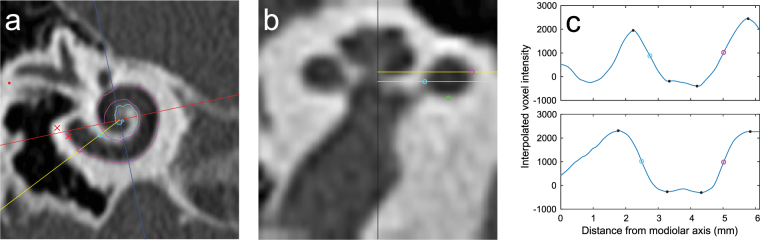
Illustration of the automatic tracing method. (**a**) MPR slice of a pre-operative cochlear CT scan. (**b**) Midmodiolar cross-section of the cochlea at the angle indicated by the yellow line in (**a**). (**c**) Interpolated voxel intensity along the yellow lines in (**a**) and (**b**); the top plot shows voxel intensities along the top yellow line in figure (**b**), while the bottom plot corresponds to the bottom yellow line shown in (**b**). The purple, light blue and green circles indicate the locations determined for the outer wall, inner wall, and floor of the scala tympani, respectively. The purple and light blue curves in the MPR of figure (**a**) are projections of the automatically traced outer and inner walls. The Black dots in figure (**c**) indicate minima and maxima.

To automatically trace the cochlear walls in pre-operative MPRs, a custom algorithm was written using MATLAB and its image processing toolbox (version R2015a). The basic principle behind the automatic tracing algorithm is illustrated in Fig. 1. Figure 1a shows an MPR slice with a coordinate system as defined in the consensus study by Verbist *et al*. (2010b). The edges of the RW are marked by red crosses (note that the edges were determined in a different MPR slice than the one depicted). The red line through the center of the RW and the modiolus of the cochlea indicates the 0°/180° cochlear angle line, and the blue line indicates the 90°/270° cochlear angle. The point where the red and blue lines intersect is the location of the modiolar axis. In this MPR, a radial line is plotted at an arbitrary cochlear angle of 25° (yellow line), along which the locations of the inner and outer walls were determined.

Figure 1b shows the corresponding midmodiolar cross-section at 25°, which is obtained by first calculating image profiles using the ‘improfile’ function in MATLAB, which interpolates the voxel intensities along the radial lines in each MPR slice (the lines were extended across the entire MPR slice and thus also included the opposite side of the modiolar axis, i.e. the radial line at 205°). Next, these image profiles were set as rows of pixels in a two-dimensional image, which was then up scaled using the ‘imresize’ function to generate the midmodiolar cross-section shown. In both the improfile and imresize functions bicubic interpolation was used and the pixel size was set equal to the voxel dimensions in the original MPR slices.

In Fig. 1b, the modiolar axis is indicated by a black vertical line at the center of the image and two radial lines, set in consecutive MPR slices which are 0.5 mm apart, are again plotted in yellow. Figure 1c shows the image profiles calculated along the radial lines shown in Fig. 1b; the top plot corresponds to the top yellow line in Fig. 1b, and the bottom plot corresponds to the bottom yellow line. In these image profiles, the minima and maxima closest to the inner and outer cochlear walls are indicated by black dots. The locations of the cochlear walls were determined in each image profile by assuming that the voxel intensity values at the boundaries are halfway between the nearest minimum and maximum intensity value; the location of the outer wall found in this manner is marked in each image profile with purple circles, while the inner wall is marked with a light blue circle.

These outer and inner wall points were determined for every MPR slice at 1 degree intervals, starting from the line through the center of the RW (0°) to a maximum angle of 720°. At each angle, the most laterally located outer wall point and the most medially located inner wall point were selected for the final traces of the outer and inner walls, respectively. In the example given in Fig. 1, the outer wall point in the top image profile of Fig. 1c was selected for the trace of the outer wall, and the inner wall point of the bottom image profile was selected for the inner wall trace; these selected points are also plotted as purple and light blue circles in Fig. 1a and b. The two-dimensional projections of the full traces of the outer and inner wall are respectively plotted as purple and light blue curves in Fig. 1a.

After the outer and inner walls were traced, the bottom walls were determined in a manner similar to the one described above. At every angle, the same method was used to interpolate voxel intensities along a line in the midmodiolar cross-section. The line of interpolation ran parallel to the modiolar axis and was located exactly halfway between the outer and inner wall points. As with the outer and inner walls, the location of the bottom wall was determined in the resulting image profile by finding the minima and maxima and assuming the bottom wall was located there where the voxel intensity was halfway between the adjacent minimum and maximum values. The green circle in Fig. 1b shows the location of the bottom wall determined by this method.

### Radial distances and vertical trajectory

To describe cochlear morphology, radial distances from the modiolar axis to the outer and inner wall were measured per angular distance from the RW to a maximum of 720 degrees (representing the basal and second turn). The averaged distance for the complete population of 479 inner ears was used to determine the average size and standard deviation (SD) as a function of cochlear angle. By subtracting the inner wall radius from the outer wall radius, the diameter of the cochlear canal was calculated. In addition, the vertical trajectory was determined for each individual patient by determining the z-coordinates of the floor of the ST relative to the center of the RW.

### Spiral fitting of cochlear walls

Cochlear shape was also described by applying a spiral fit through the radial distances of the inner and outer walls separately. From observations, an assumption was made that the outer and inner wall radial distances did not completely follow a logarithmic spiral function, but remained more or less constant from halfway through the second turn towards the apical region. Therefore, we introduced an offset value into the logarithmic spiral function that is commonly used to describe the cochlear curve^5,7^. Subsequently, the curve that optimally represents the 720 radial distances of the outer and inner walls to the center of the modiolus was fitted by an individually adjusted exponential fitting formula based on the curve of a logarithmic spiral, both without (equation 1) and with (equation 2) an offset value.

1 $${\rm{r}}(\ominus )={{\rm{ae}}}^{{\rm{b}}\ominus }$$

2 $${\rm{r}}(\ominus )={{\rm{ae}}}^{{\rm{b}}\ominus }+{\rm{c}},$$

where r represents the distance to the center of the modiolus, $\ominus$ the corresponding angular distance, a and b the coefficients, and c the offset value. These fitting formulas were applied to each inner ear individually, as well as over the median measurements per angle of the 479 inner ears. The fit of the radial distances of the inner and outer walls, indicated by the root mean square (RMS), was compared between the function with and without an offset value.

### Manually versus automatically determined cochlear walls

In a previous study at our institution, the outer and inner walls of the basal turn of the cochlea were determined manually by scrolling through the pre-operative MPRs, to search their boundaries^5^. Four outer wall radii, four inner wall radii, and four cochlear duct sizes were determined at 0, 90, 180, and 270 degrees in accordance with the consensus on cochlear coordinates (Verbist, 2010). This comparison could only be made for the basal turn because only the automatically measurements extend beyond the basal turn. The measurements of the inner and outer radii were compared to the output from the automatically traced values using a paired samples t-test.

### Data availability

The datasets generated in this study are available from the corresponding author on reasonable request.

## Results

### Method applicability

Tracing the complete two turns could be achieved in respectively all 479 for the outer wall and 192 cochleas for the inner wall. In the remaining 287 cochleas, the inner wall was traced up to 577 degrees on average (SD 161).

### Radial distances and cochlear canal size

**Figure 2:**
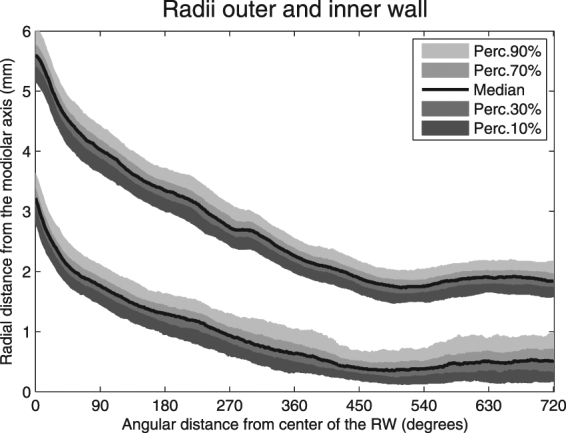
Automatically traced radial distances from the outer (upper lines) and inner (lower lines) walls to the modiolar axis from a cochlear angle of 0 to 720 degrees.

**Figure 3:**
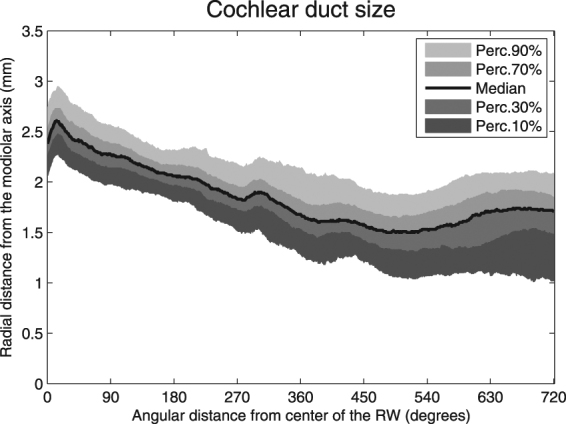
Diameter of the cochlear duct from 0 to 720 degrees.

Figure 2 shows the median, 10^th^, 30^th^, 70^th^, and 90^th^ percentile radii of the outer (upper lines) and inner (lower lines) walls, automatically traced from the center of the RW in the apical direction. The radii decreased gradually until 500 degrees for both the outer and inner walls. Between 500 and 720 degrees, the inner and outer walls do not further approximate the modiolus or each other; the radii slightly increase in size from there, the outer wall more so than the inner wall. The smallest median outer wall radius was measured at 516 degrees (1.74 mm). From this minimum in the apical direction, the largest outer wall radius was measured at 645 degrees (1.90 mm) (p < 0.001). For the inner wall, the shortest radius (0.39 mm) was measured at 492 degrees. More apical, the maximal radial distance of the inner wall to the modiolus was 0.53 mm at 619 degrees (p < 0.001). The limited decrease in the radial distances is also reflected in the size of the cochlear duct calculated from the median values for the outer and inner walls (Fig. 3). At the basal portion, from 0 to 15 degrees, the median diameter of the cochlear duct gradually grows to a maximum of 2.6 mm. From 15 degrees, the lumen decreases gradually to a minimum of 1.5 mm at 500 degrees. Around 270 degrees, the radius of the outer wall remains more or less stable for approximately 30 degrees. This pattern is not present in the inner wall measurements. Thus, a slight decrease in the cochlear duct size is visible around that area. The smallest cochlear duct size was found around 500 degrees. From 555 degrees, the median cochlear duct size slightly expands to a maximum size of 1.7 mm at 662 degrees. The slightly irregular tapering of both the basal and second turn is clearly visible in Figs 2 and 3. Also, an increasing and uneven change in both radial distance and cochlear duct size from the basal end to the end of the second turn is evident. The median outer wall length for two complete cochlear turns was 35.1 mm (range 30.3–41.5 mm) in the entire population of 479 ears. The inner wall in 192 inner ears had a median length of 15.9 mm (range 10.6–24.8 mm).

### Spiral fitting of cochlear walls

**Figure 4:**
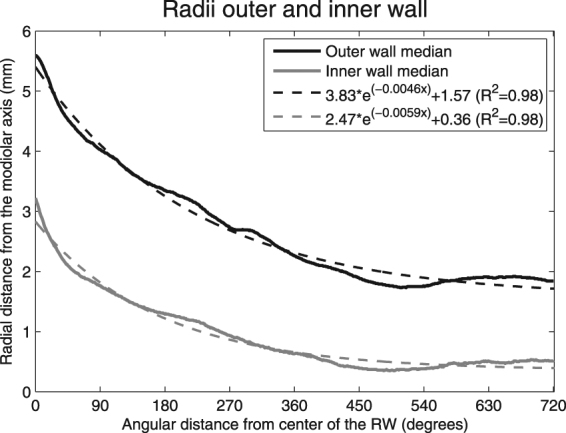
Median radial distances of the outer (red) and inner walls (blue) from the central axis of the modiolus. The dashed lines indicate the spiral functions.

Figure 4 shows the median radial distances from the modiolar axis to the outer (red line) and inner (blue line) walls of the cochlea from 0 towards 720 angular degrees. The graph shows an exponential reduction in the radial distance for the first approximately 500 degrees (1.4 turn), but beyond the length of the radii it remains almost constant. This suggests that an offset value needs to be added to the logarithmic spiral function in order to more veraciously model the course of the cochlear duct. Using logarithmic transformation and regression analysis, spiral fitting formula coefficients were determined for the radial lengths of the inner and outer walls for each individual inner ear, both with and without the implementation of an off-set value. With the implemented off-set value, the accuracy of the spiral function (R^2^) to describe the radial distances increased from 0.90 (SD 0.05) to 0.96 (SD 0.04) for the outer wall (p < 0.001) and from 0.88 (SD 0.15) to 0.91 (SD 0.09) for the inner wall (p < 0.001). The median outer and inner wall radial distances (solid lines) in 479 inner ears and its logarithmic spiral function with off-set value (dashed lines) are shown in Fig. 4.

### Floor of the scala tympani

**Figure 5:**
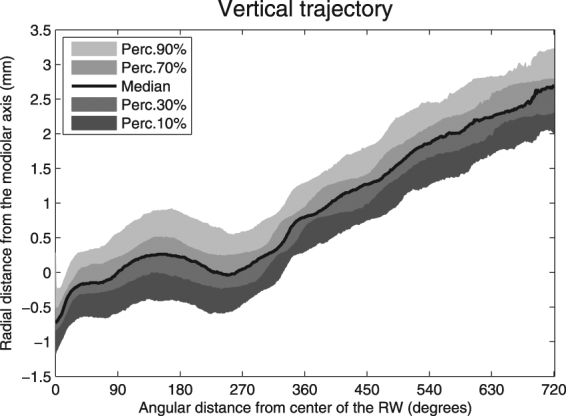
Automatically traced vertical trajectory of the floor of the scala tympani.

**Figure 6:**
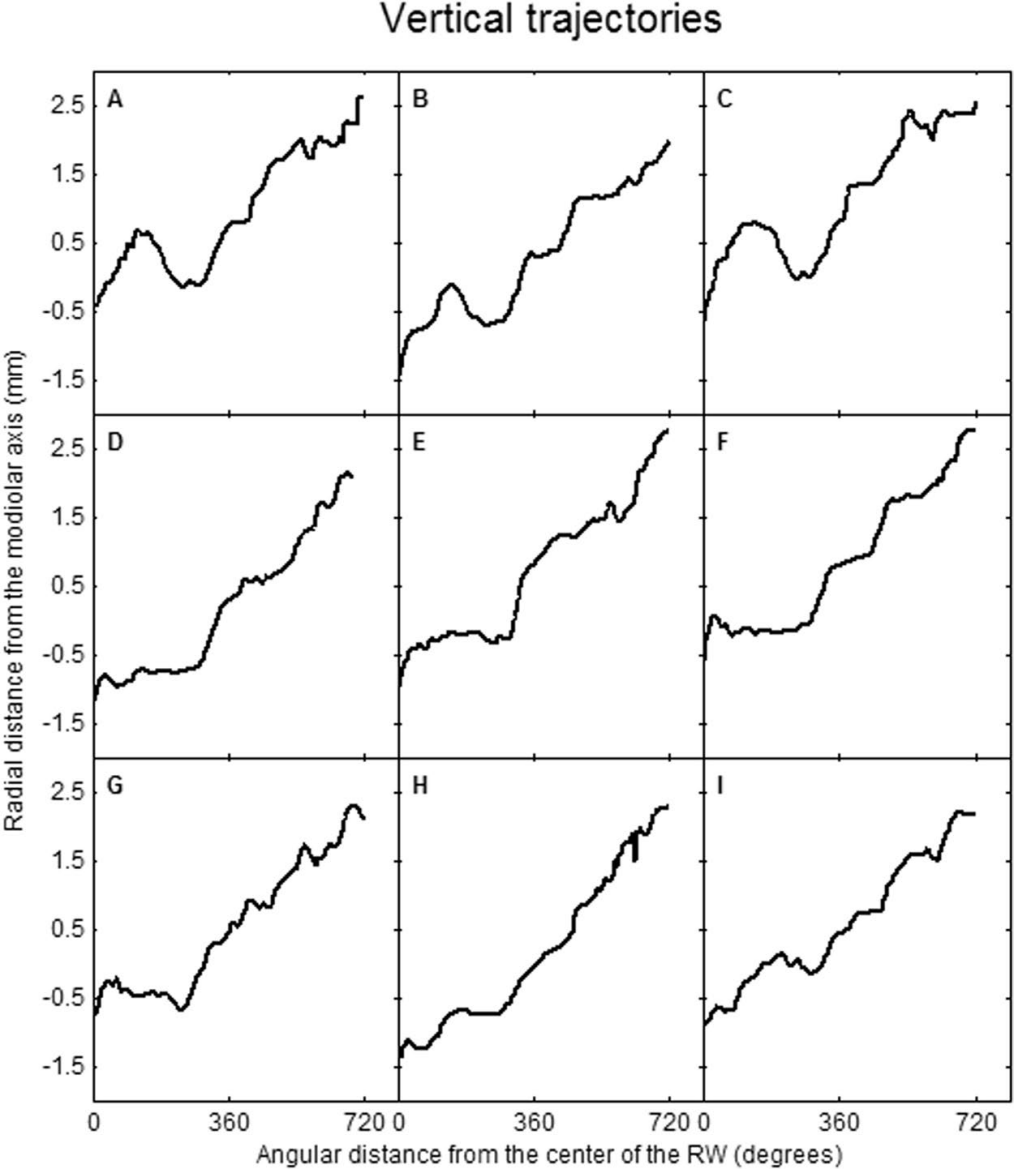
Examples of automatically traced vertical trajectories of the floor of the scala tympani showing a proximal rise and fall within the basal turn (**A**–**C**) and late steep rising, with a preceding fall or flat course (**D**–**F**) or preceding fall (**G**) within the first 360 degrees, or a more or less constant rising of the vertical slope (**H**–**I**).

Figure 5 represents the median vertical trajectory of the floor of the ST, as well as the 10^th^, 30^th^, 70^th^, and 90^th^ percentiles. The figure shows that, especially in the first 270 degrees, the vertical trajectory varies greatly. The least variation in the vertical trajectory is present at the confluence of the first and second turns. To illustrate the large variation, an overview of nine individual trajectories is presented in Fig. 6. Roughly three different courses of the basal turn can be distinguished. Course 1 is a proximal rise and fall within the basal turn (Fig. 6A–C). Course 2 is a late steep rising, with a preceding flat course (Fig. 6D–F) or fall (Fig. 6G) within the first 360 degrees. Course 3 has a more or less constant rising of the vertical slope (Fig. 6H and I).

### Manually versus automatically determined cochlear walls

The outcomes of the manually and automatically determined radial distances are presented in Table 2. Significant differences between them were found for all measurements. For the outer wall at 0, 90, and 180 angular degrees from the RW, radial distances were larger when measured automatically. At 270 degrees, the automatic measurements obtained a smaller radial distance. A smaller radial distance was measured for the first three radii of the inner wall when measurements were performed automatically. The fourth inner wall radius, at 270 degrees, was larger when determined automatically. For both methods the mean paired difference and standard error of the mean increased along the cochlear duct toward the more apical measurements.

**Table 2:** Manually versus Automatically determined radial distances.

Manually			Automatically	Mean diff	Std. error mean	p-value	Effect size (Cohen’s D)
Outer wall	Degrees	Mean (mm), *SD*	Mean (mm), *SD*				
1	0	5.60, *0.39*	5.62, *0.40*	−*0.021*	*0.001*	*0.037*	*0.05*
2	90	3.96, *0.31*	4.01, *0.30*	−*0.042*	*0.012*	<*0.001*	*0.16*
3	180	3.33, *0.29*	3.35, *0.30*	−*0.014*	*0.012*	*0.032*	*0.07*
4	270	2.81, *0.29*	2.77, *0.27*	*0.064*	*0.013*	<*0.001*	*0.14*
**Inner wall**							
1	0	3.41, *0.31*	3.23, *0.33*	*0.177*	*0.009*	<*0.001*	*0.58*
2	90	2.03, *0.28*	1.76, *0.26*	*0.283*	*0.012*	<*0.001*	*0.96*
3	180	1.45, *0.27*	1.29, *0.28*	*0.309*	*0.014*	<*0.001*	*0.59*
4	270	0.94, *0.23*	0.95, *0.27*	*0.290*	*0.013*	*0.002*	*0.04*

## Discussion

This study is the first to measure cochlear morphology *in vivo* up to and including the second turn of the cochlea using an automatic tracing method based on voxel intensity. The greatest number of inner ear measurements is involved for describing the morphological characteristics of the human cochlea. This method allows *in vivo* evaluation of cochlear morphology, providing valuable insight into individual, patient-specific anatomical features and its variability on a large scale.

An important aspect of our study is the inclusion of the second turn of the cochlea to describe cochlear morphology *in vivo*. Although a few studies have reported on the variable dimensions of the second turn *ex vivo*, evaluation of the second turn in clinical practice has been very limited described^2,3,10–12^. Würfel *et al*. introduced a methodology to determine individual cochlear length along the complete lateral wall from clinical Cone Beam CT scans, providing an indication of cochlear size. However, the inner walls were not determined in their study and therefore the ability to determine the diameter of the cochlear duct is lacking. Distinction of, particularly, the inner walls beyond the basal turn is challenging because of the increased noise caused by the modiolus. However, including the second turn in the pre-operative evaluation may be valuable, as the majority of CI designs have a target insertion depth over 360 degrees. Van der Marel *et al*. demonstrated a surgical guidance model based on details of cochlear morphology derived from preoperative CT scans^13^. Optimal surgical insertion distance, indicated by the linear distance between the round window and most basal electrode contact, was studied in order to target a specific angular insertion depth. They tested two different methods; the spiral fit and the multiple regression method and found better results with the linear regression model including three input parameters. The poorer prediction of the spiral fit model was explained by the fact that only the radical distances of the basal turn were available and implemented in the model to fit the spiral. This results in extrapolation from these known values, as the angular insertion depth is frequently located beyond the basal turn. This leads to less accurate estimation of the actual angular insertion depth. Our method allows inclusion of measurements of the second turn in order to hopefully achieve a better spiral fit and improvement of the spiral model for surgical guidance.

Theoretically, the algorithm we described could trace the complete cochlea, but this would likely introduce inaccuracies due to impaired depiction of the third turn. The diameter of the cochlear duct tapers substantially along its course towards the apical region where a mismatch with the diameter of the electrode array may occur^1,14–16^. The ability to measure the variability of the cochlear diameter up to 720 degrees on a large scale may be used to predict which cochleas are at risk for traumatic insertions. We showed that this narrowing pattern does not follow a gradual course along 720 degrees. Earlier studies already reported irregular narrowing of the cochlear canal in the first 360 degrees^5^. A second valuable aspect of our study is the number of measurements used to determine cochlear size and shape. In previous studies, it was common to express the diameter of the cochlea in measurements along two, four, or eight radii^5,6,17^. Using 721 assessments, one per angular angle, the tapering course of the cochlear walls can be mapped more accurately.

An interesting outcome of our study was the stable radial distance between approximately 500 and 720 degrees, of both the outer and inner walls. This has not been described previously in the literature, though it was previously noted by Prof. A. Kral at the conference on Cochlear Implants and other Auditory Prostheses (CIAP) in July 2015^18^. The shape of the cochlea is most often described on a logarithmic scale with exponentially decreasing radii^5,7^. However, in our study, adding an off-set value to the logarithmic function improved the fit of the cochlear shape to the logarithmic function.

The slope of the vertical trajectory is likely to influence the course of the electrode array and we predict a high risk of damaging the basilar membrane or osseous spiral lamina where the slope shows a steep in- or decrease. Such a decrease is generally present near the confluence of the first and second half of the basal turn, around 180 degrees^1,8^. Avci *et al*. demonstrated the considerable variability in the vertical slope of the cochlea and the impact of cochlear geometry on insertion forces^1,19^. They studied micro-CT scans of temporal bones and applied a risk profile based on the presence of so-called dips, peaks, and vertical jumps, indicating significant changes in the vertical trajectory^1^. Our measurements showed a change in steepness or direction of the slope at four specific locations in the basal turn (Fig. 4). Taking the large variability shown in Fig. 5 into account, our method allows *in vivo* classification of slope characteristics and can be used to estimate the risk of insertion trauma.

Comparing manual and automatic measuring, the outer wall radii at 0, 90, and 180 degrees from the RW are smaller when determined manually, where the inner wall radii are larger at these three locations. Contrarily, the outer and inner wall radii at 270 degrees are smaller and larger, respectively, when manually determined. This finding can be explained by the difference in the voxel density of the aligning structures. Near the inner wall, the soft tissue within the modiolus hampers detection of the contours of the inner wall. The fact that the small differences reach significance for all eight outer and inner wall radii can presumably be explained by the large dataset. All automatically derived measurements are more consistent with micro-CT data from temporal bones^1^. In addition, two other studies, using plastic casts from unselected temporal bone specimen, reported measurements more consistent with our automatically derived measurements for all radii except the large diameter in 1 study^2,4^. However, our results only demonstrate the differences between the manual and automatic measurement, without the ability to make a ruling which of the two methods approached reality the most. Ideally, a comparison must be made between our measurements, both manually and automatically, and measurements collected from histological preparations, but for obvious reasons this is not possible. However, we believe that the automatically measured ones are more accurate and less susceptible for observer variation because of the use of 180 measurements per cochlear turn instead of 4 and because an objective instead of subjective method is being used.

In this study, the dimensions of the cochlea were determined from automatically traced measurements of the outer and inner wall and the bottom of the ST. Within the cochlear duct, no distinction between ST and scala vestibuli (SV) was made. Previous studies demonstrated that the dimensions of both the ST and SV are not evenly distributed over the cochlear duct, and the shapes of both scalae change along the course^20–23^. Biedron *et al*. and Wysocki *et al*. showed that the size of the ST is more prominent than the SV during the first half of the basal turn^22,23^. The diameter of the cochlear duct has been shown to represent the diameter of the ST during this initial trajectory. Subsequently, the SV dominates the ST in size and is the size of the cochlear duct, not representative of the actual dimension of the ST^24^. A more distinctive depiction of the osseous spiral lamina is required to define scalar dimensions. Unfortunately, this is not achievable with current *in vivo* imaging techniques, particularly for the second turn, due to the limited spatial resolution of CT images. MRI may approximate the actual boundaries of the ST more accurately than CT. As CT shows the bony limit of the outer wall, MRI reveals the inner limit of the outer wall at the confluence of the perilymph in the ST to the outer wall. Using MRI may also overcome the challenges associated with measuring the inner wall. Nevertheless, this study was conducted using CT images because pre-operative evaluation of CI candidates is carried out using only CT in most institutions. In the near future, a study will be conducted in the near future to compare CT and MRI using our method.

## Conclusion

In conclusion, the automatic method we introduced here offers an objective way of determining the morphology of the basal and second turn of the cochlea. We demonstrated that the shape of the cochlear duct does not follow a pure logarithmic spiral function. An additional offset value to the function further approximates the true shape of the cochlear duct. The outcomes of our study can be used in a clinical setting as part of the pre-operative evaluation of CI candidates. Our results also provide valuable insight into the variability of individual human cochleas, especially the vertical trajectory and cochlear canal size.
